# Heart Transplantation for Congenital Heart Disease in the First Year of Life

**DOI:** 10.2174/157340311797484231

**Published:** 2011-05

**Authors:** Richard E Chinnock, Leonard L Bailey

**Affiliations:** 1 Departments of Pediatrics Loma Linda University School of Medicine Pediatric Heart Transplant Program Loma Linda University Children’s Hospital Loma Linda, CA, USA; 2 Departments of Surgery Loma Linda University School of Medicine Pediatric Heart Transplant Program Loma Linda University Children’s Hospital Loma Linda, CA, USA

**Keywords:** Clinical outcome, congenital heart disease, donor issues, infant heart transplant, hypoplastic left heart syndrome, indications for transplant.

## Abstract

Successful infant heart transplantation has now been performed for over 25 years. Assessment of long term outcomes is now possible. We report clinical outcomes for322 patients who received their heart transplant during infancy. Actuarial graft survival for newborn recipients is 59% at 25 years. Survival has improved in the most recent era. Cardiac allograft vasculopathy is the most important late cause of death with an actuarial incidence at 25 years of 35%. Post-transplant lymphoma is estimated to occur in 20% of infant recipients by25 years. Chronic kidney disease grade 3 or worse is present in 31% of survivors. The epidemiology of infant heart transplantation has changed through the years as the results for staged repair improved and donor resources remained stagnant. Most centers now employ staged repair for hypoplastic left heart syndrome and similar extreme forms of congenital heart disease. Techniques for staged repair, including the hybrid procedure, are described. The lack of donors is described with particular note regarding decreased donors due to newer programs for appropriate infant sleep positioning and infant car seats. ABO incompatible donors are a newer resource for maximizing donor resources, as is donation after circulatory determination of death and techniques to properly utilize more donors by expanding the criteria for what is an acceptable donor. An immunological advantage for the youngest recipients has long been postulated, and evaluation of this phenomenon may provide clues to the development of accommodation and/or tolerance.

## HISTORICAL PERSPECTIVE

Infant heart transplantation was first attempted by Adrian Kantrowitz 3 days after Christiaan Barnard’s famous first transplant performed in 1967. Magdi Yacoub in London performed a heart transplant on a 10-day-old baby in 1984. The infant survived 18 days. In the fall of the same year, Leonard Bailey and the team at Loma Linda University performed a xenotransplant operation, inserting the heart of a baboon into a 12-day-old baby who survived 20 days. The first successful infant recipient was transplanted by Denton Cooley in 1984 and this child survived 13 years. The first successful newborn heart recipient was transplanted at Loma Linda in 1985 at the age of 4 days. He is still alive and well, with his first graft, 25 years later [1].

There have been 8575 pediatric heart transplant procedures reported to the International Society for Heart and Lung Transplantation Registry through 2009 [2]. Of these, 2171 (25%) have been performed in infants. The number of infant heart transplants reported per year has remained relatively stable over the last decade at approximately 100 procedures per year. Over the years, though, the proportion of transplant procedures performed for infants with a diagnosis of congenital heart disease has changed from 80% of the total in 1988-1995 to 63% from 1996-2009 [3].

In the last quarter century, much has been learned about infant heart transplantation. But, much remains to be discovered. This report will describe the clinical outcome of infant heart transplant recipients, utilizing the Loma Linda experience, concentrating on survival, rejection, cardiac allograft vasculopathy, post-transplant lymphoproliferative disease and long-term renal function. In addition, we will describe issues that are especially pertinent to infant heart transplantation; donor availability, ABO incompatible transplantation, surgical management of infants with hypoplastic left heart syndrome (HLHS) and similar extreme forms of congenital heart disease, and transplantation and the hybrid procedure for palliation of HLHS.

## CLINICAL OUTCOME OF INFANT HEART TRANSPLANTATION – THE LOMA LINDA EXPERIENCE:

**Fig. (1):**
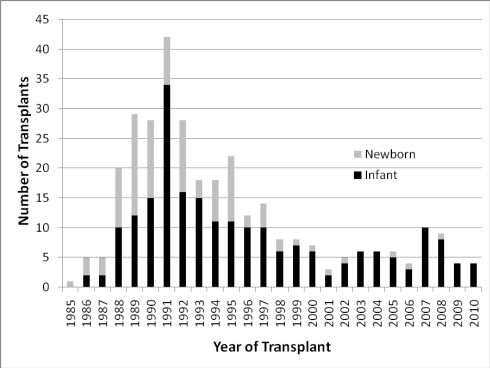
Number of patients transplanted in each year, categorized by Newborn (First 30 days) or Infant (31-365 days).

There have been 322 infant heart transplant procedures performed at Loma Linda University Children’s Hospital from November 1985 through November 2010. Of these, 103 or 32 % were performed during the first month of life, with the youngest transplanted at 1.5 hours of life [4]. Early in the experience, the indication for the vast majority of these transplants was HLHS. Because of limited donor availability, most newborns with HLHS now receive staged surgical repair. Fig. **1** illustrates the number of transplant procedures in newborns (0-30 days) and infants (1-12 months) by year. One can see that the proportion of newborns transplanted has declined, illustrating the change in donor availability over time.

**Fig. (2):**
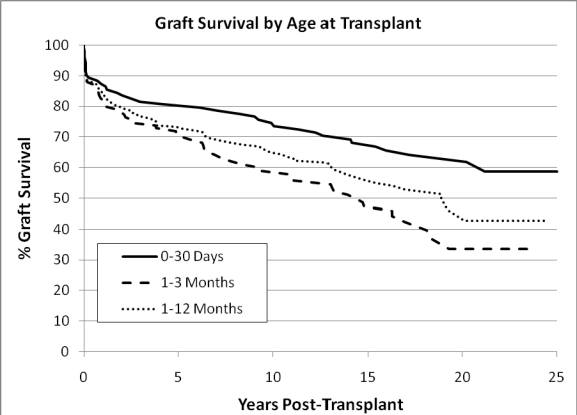
Actuarial graft survival. Log rank p=0.027 for 0-30 day old recipients versus 1-3 month old recipients

The actuarial survival for these patients is illustrated in Fig. **2**. Newborn recipients have better survival than those transplanted later in infancy. The reason for this is probably multi-factorial and likely includes an immunological advantage to early transplant (discussed below) and avoidance of complications that arise with prolonged waiting time. The graft half-life for newborn recipients is still undefined, with an actuarial graft survival at 25 years of 59%, likely the longest graft half-life for any solid organ transplant population.

**Fig. (3):**
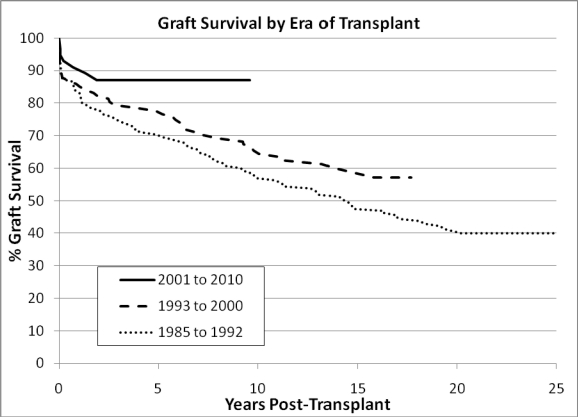
Graft Survival by Era of Transplant. (Kaplan-Meier analysis; p=0.02 by log rank).

There has been an era effect similar to effects reported in the ISHLT registry report. This is illustrated in Fig. **3**.

The causes of death are similar to what one might expect, with acute and chronic rejection (i.e. cardiac allograft vasculopathy - or CAV) being the most significant [5]. Acute rejection accounted for 16% of deaths, acute rejection with accompanying CAV accounted for 8% of deaths, and CAV alone accounted for 14% of deaths. Acute graft dysfunction was only present in 5 infant recipients. Infection caused 16% of deaths and neoplasms, most commonly post-transplant lymphoproliferative disease, accounted for 9% of deaths. 

Our immunosuppression regimen has evolved over the years, but it has consistently had the goal of steroid avoidance. Less than 5% of our infant recipients require maintenance steroids to prevent rejection. Similar shorter term outcomes have recently been demonstrated by the Boston Children's group [6]. Our current protocol is included in the Appendix.

**Fig. (4):**
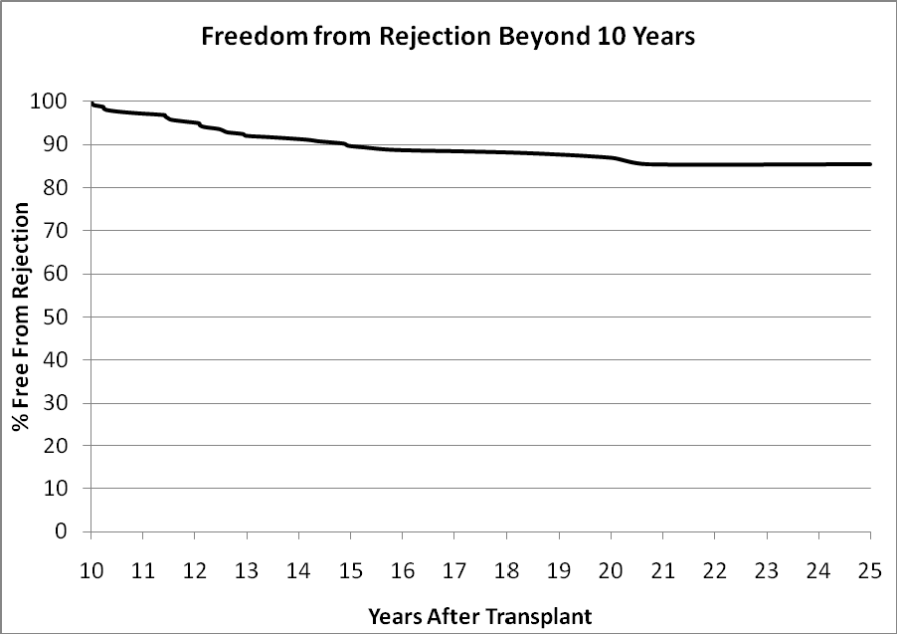
Freedom from rejection starting at 10 years after transplantation. N=265.

Acute rejection late after infant heart transplantation occurs, as illustrated by Fig. **4**. Between 10 and 20 years after infant heart transplantation approximately 30% of patients will experience a rejection episode. There is a particular hazard of rejection during adolescence when issues of non-adherence become more important.

**Fig. (5):**
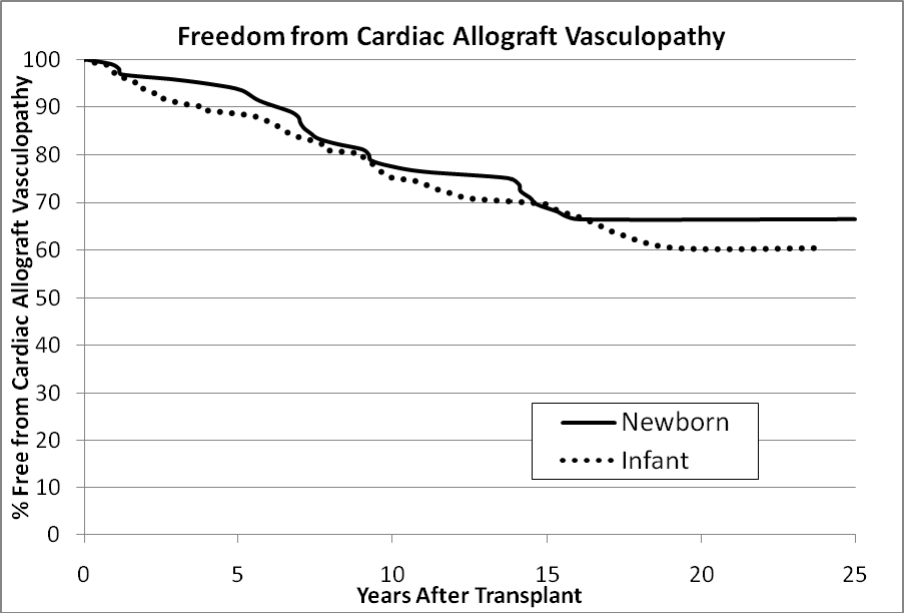
Freedom from cardiac allograft vasculopathy (CAV) by age at transplant. Newborn = 0-30 days, Infant = 31-365 days. No statistical significant difference, p=0.495 by log rank test.

Consistent with other reports, [3] cardiac allograft vasculopathy is the greatest barrier to long-term graft and patient survival. The ISHLT registry report notes that the 10 year freedom from CAV in the infant population was 73%. They also note that not being on steroids at the time of discharge may decrease the risk of CAV (RR 0.61; p=0.07). It is possible that our steroid avoidance regimen contributes to our 10 year freedom from CAV rate of 82%. The 25 year actuarial freedom from CAV in our infant population is illustrated in Fig. **5**.

**Fig. (6):**
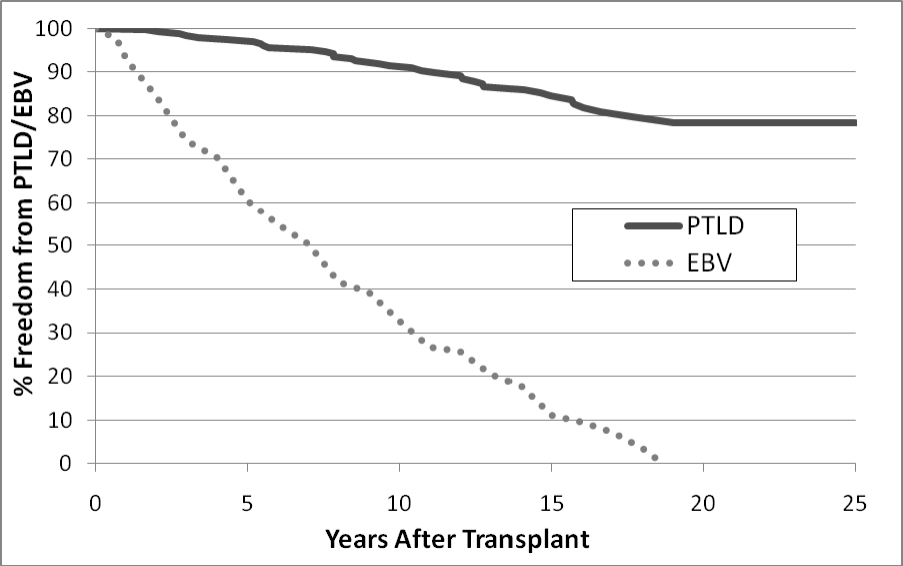
Actuarial freedom from PTLD after infant heart transplantation (solid line); Earliest evidence of infection (either by serology or Polymerase Chain Reaction) with EBV (dotted line).

Post-transplant lymphoproliferative disease (PTLD) is another important cause of late morbidity and mortality. In a multi-center report [7], the probability of PTLD was reported as 8% at 5 years post-transplant. A single center report [8] described an actuarial risk of PTLD at 10 years at 28%. In our infant population we have seen a 22% actuarial risk of PTLD at 25 years after transplant (see Fig. **6**). Interestingly, the time to infection parallels the risk of PTLD with essentially all infant recipients predicted to have been infected by Ebstein Barr Virus (EBV) by 19 years after transplant. (Also see Fig. **6**)

**Fig. (7):**
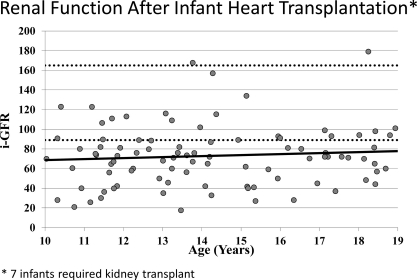
Most recent i-GFR represented, each dot represents an individual patient. Upper and lower limits of normal (dotted line); trend line (solid line).

Long-term renal function is a particular concern in a population that is hoped to survive for decades after transplant. It is a particular concern in the infant population since they are transplanted before kidney maturity has occurred. Of those infants who have survived at least 10 years, 47% are receiving anti-hypertension therapy. We have also undertaken a program of thorough evaluation of renal function, performing an isotopic glomerular filtration rate (i-GFR) on an annual basis on all patients followed at our institution. The most recent i-GFR for 93 infant recipients who have survived at least 10 years is noted in Fig. **7**. The average i-GFR in these patients is 73 mL/min/1.73m^2^. However, there are a significant number of patients with significant renal insufficiency and 7 infants have received renal replacement therapy late after heart transplantation. The number of patients in each category of chronic kidney disease (as defined solely by their GFR) is illustrated in Table **1**.

## CLINICAL ISSUES IN INFANT HEART TRANSPLANTATION

There is now a 25 year history of successful infant heart transplantation. Much has been learned, but much remains to be understood. The following will describe those issues most applicable to infant heart transplantation:

- Which congenital heart lesions should be managed with heart transplantation as primary therapy, if any?
- What is the optimal approach to hypoplastic left heart syndrome and equivalent malformations?
- How might the supply of donors be improved?
- Is there an immunologic advantage for the younger recipient?

### Surgical Management of Infants with Hypoplastic Left Heart Syndrome and Similar Extreme Forms of Congenital Heart Disease

Hypoplastic left heart syndrome (HLHS) was once considered beyond surgical relief. Infants born with it, died because of it. During the past quarter-century, however, the story has dramatically improved. Norwood’s staged-reconstruction and infant heart transplantation grew out of the early 1980’s to create a positive change in survival for infants with this and similar extreme forms of congenital heart disease [9, 10]. Neither treatment pathway threatened development of the other. The need for effective and reproducible therapy was just too great. Oddly enough, it was a significant early difference in operative survival that gave both strategies momentum. During the 1980’s and early 1990’s, survival following transplantation was the rule, whereas survival was more the exception after Norwood’s procedure. This differential in outcomes not only served to establish transplantation as an important, if limited, option, but it encouraged Norwood and a handful of other skillful and committed surgical teams toward improved infant survival following palliative-reconstruction. Eventually, donor offers plateaued (never more than about 100 annually in North America), and survival following reconstruction became remarkably competitive [11-13]. By the mid to late 1990’s, virtually every center, Loma Linda included, had shifted to Norwood’s strategy as primary therapy for HLHS and equivalent cardiac anomalies. Heart transplantation, because of donor limitations, became consigned, as primary therapy, to those very few infants deemed unsuitable for staged-reconstruction. Primary transplantation has remained available in some centers as a parental choice, and as the only solution for the occasional young infant with profound cardiomyopathic disease, including some tumors.

**Fig. (8):**
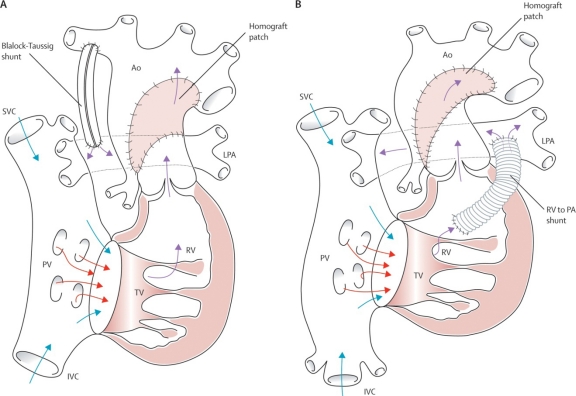
**A/B**. Norwood’s procedure for hypoplastic left heart syndrome and equivalent malformations. (**A**) The classic procedure using a modified Blalock-Taussig shunt, and (**B**) the Sano modification using a conduit from ventricle to pulmonary arteries. Ao=aorta. IVC=inferior vena cava. LPA=left pulmonary artery. PV=pulmonary valve. PA=pulmonary artery. RPA=right pulmonary artery. RV=right ventricle. SVC=superior vena cava. TV=tricuspid valve. Barron DJ, Kilby MD, Davies B, Wright JGC, Jones TJ, Brawn WJ. Hypoplastic left heart syndrome. Lancet 2009; 374:551-64. Used with permission of the publisher.

Both understanding and application of Norwood’s staged-reconstruction has evolved brilliantly during the past two decades. The approach now includes three separate stages. The first, and most challenging stage is accomplished in the neonate. It involves aortic arch reconstruction (usually with pulmonary allograft tissue) and uses the native pulmonary valve for systemic ventricular outflow. The pulmonary circulation is separated from the heart and is driven by means of a modified Blalock-Taussig shunt (Fig **8-A**). Alternatively, a polytetrafluoroethylene (PTFE) tube is used to connect the base of the ventricle to the branch pulmonary arteries, a modification introduced by Sano and colleagues (Fig. **8-B**) [14]. The atrial septum is excised.

As many as 9 out of 10 neonates now survive Norwood’s first stage of reconstruction. Survival following stage-one reconstruction appears to be independent of the source of pulmonary blood flow; i.e., Blalock-Taussig shunt vs. Sano conduit, but many believe that perioperative management is less stressful for infants who have Sano’s modification. While there remains some interstage attrition, the vast majority of these babies reach consideration for a second stage procedure.

**Fig. (9):**
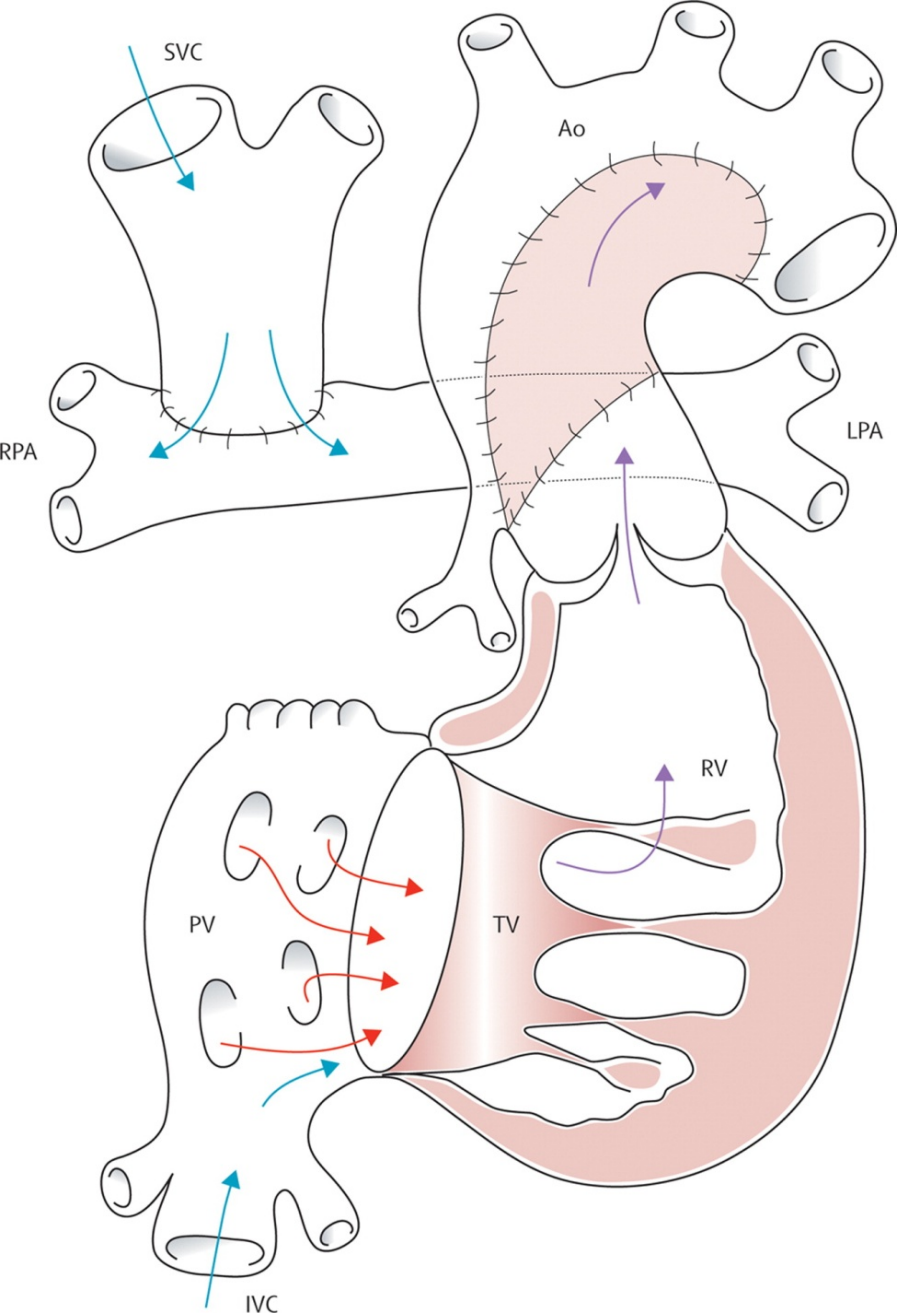
Cavopulmonary (Glenn) anastomosis – the second stage of palliative reconstruction. Ao=aorta. IVC=inferior vena cava. LPA=left pulmonary artery. PV=pulmonary valve. RPA=right pulmonary artery. RV=right ventricle. SVC=superior vena cava. TV=tricuspid valve. Barron DJ, Kilby MD, Davies B, Wright JGC, Jones TJ, Brawn WJ. Hypoplastic left heart syndrome. Lancet 2009; 374:551-64. Used with permission of the publisher.

The second stage of reconstruction is applied between four and eight months of age and requires adequate preoperative physiology in the form of low pulmonary pressure and resistence, good ventricular and atrioventricular valve function. Stage-two varies depending upon the projected form the final Fontan stage will take. If an intra-atrial lateral tunnel or if a catheter-based completion of the Fontan circuit is anticipated, then stage-two reconstruction usually consists of a so-called “hemi-Fontan” procedure. This involves interruption or takedown of the first-stage source of pulmonary blood flow and creation of bidirectional superior vena caval-to-pulmonary artery flow that incorporates the superior pole of the right atrium. The atrial cavity is separated from the pulmonary circulation by means of a thin baffle of tissue or PTFE. This sets the stage for intra-atrial lateral tunnel Fontan completion. If catheter-based completion is anticipated, a short circular tube of PTFE is sutured around the inferior vena caval entrance to the atrium. This becomes an attachment site for a covered stent extending from the inferior vena cava to the pulmonary artery. Alternatively, the second stage procedure is nothing more than takedown of the previous source of pulmonary blood flow and creation of a bidirectional cavopulmonary (Glenn) anastomosis (Fig. **9**). This is the procedure used at Loma Linda. Any additional aortic arch reconstruction or atrioseptectomy is accomplished at the second stage.

**Fig. (10):**
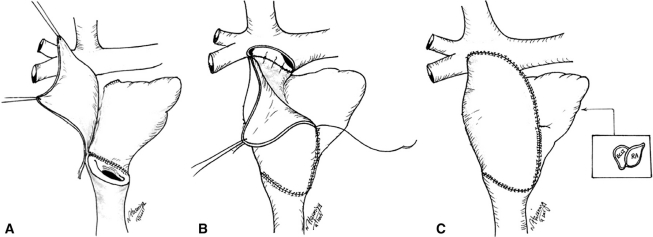
The Fontan completion as accomplished at Loma Linda University Children’s Hospital. A-C, surgical technique. An extracardiac tunnel is fashioned from in situ (pedicled) autologous pericardium. ECLT=extracardiac lateral tunnel, RA=right atrium. Hasaniya NW, Razzouk AJ, Mulla NF, Larsen RL. In situ pericardial extracardiac lateral tunnel Fontan operation: Fifteen-year experience. J Thorac Cardiovasc Surg 2010; 140: 1076-83. Used with permission of the publisher.

Timing of the final or third stage varies depending upon the anticipated type of Fontan completion. Many centers, including Loma Linda, complete the final stage of reconstruction between 18 and 24 months of age. A pedicled tube of autologous pericardium is utilized for extracardiac Fontan completion at Loma Linda (Fig. **10**) [15]. 

**Fig. (11):**
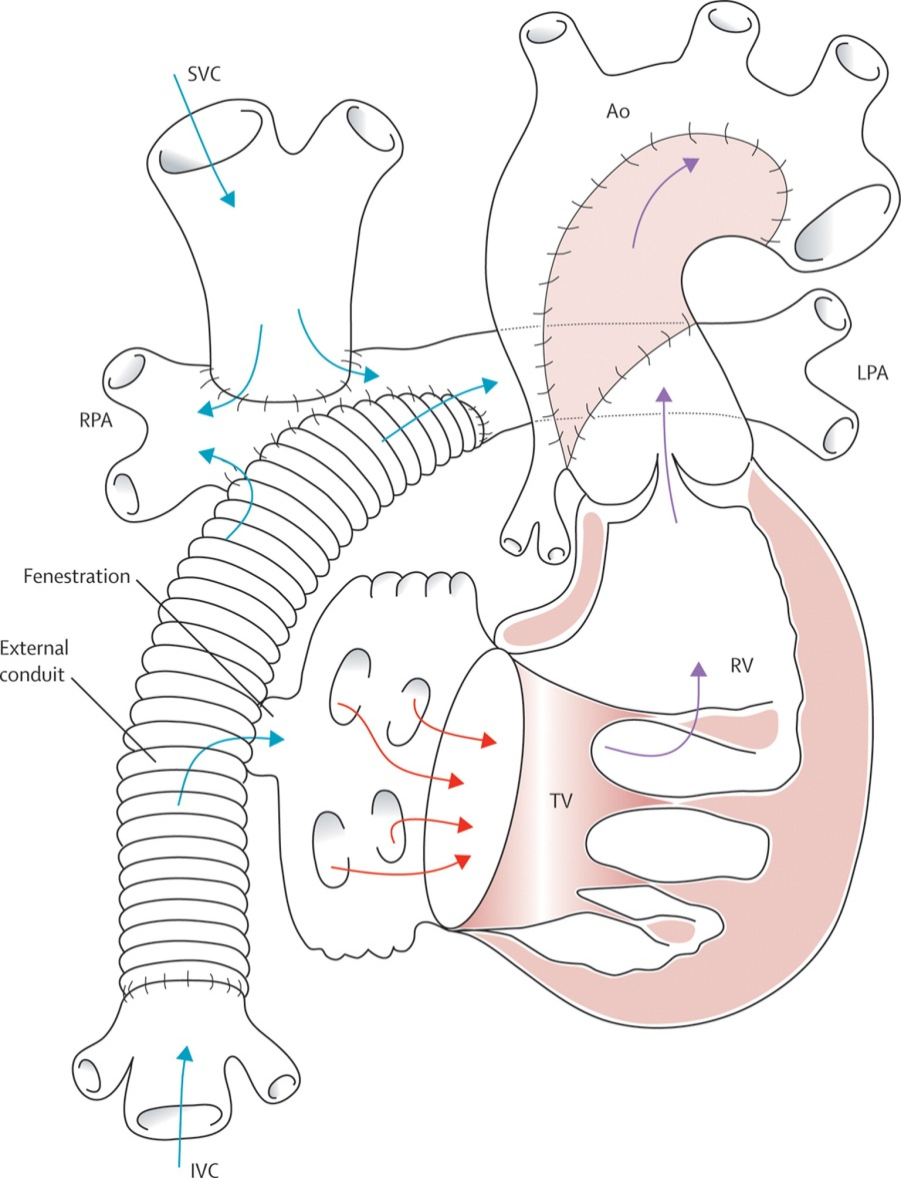
Total cavopulmonary connection – the stage-three Fontan completion. An extracardiac prosthetic conduit is utilized to carry inferior vena caval flow to the pulmonary arteries. Ao=aorta. IVC=inferior vena cava. LPA=left pulmonary artery. RPA=right pulmonary artery. RV=right ventricle. SVC=superior vena cava. TV=tricuspid valve. Barron DJ, Kilby MD, Davies B, Wright JGC, Jones TJ, Brawn WJ. Hypoplastic left heart syndrome. Lancet 2009; 374: 551-64. Used with permission of the publisher.

Those centers using an extra-cardiac prosthetic conduit may choose to delay the final stage to age three or four in an effort to accommodate a larger inferior vena cava-to-pulmonary artery conduit (Fig. **11**). 

An infant with a hemi-Fontan circuit will have an intra-atrial lateral tunnel constructed of PTFE. The stage-two baffle separating the pulmonary circuit from the atrium is, of course, excised. The issue of routine fenestration in the Fontan circulation remains open to debate. Fenestration has rarely been employed in the Loma Linda experience. Catheter-based completion of the Fontan circuit has, thus far, failed to gain traction for lack of appropriate devices.

While outcomes of staged reconstruction in the present era have become truly spectacular in experienced centers, still a few infants and children fail to respond satisfactorily [16]. Transplantation has occasionally become necessary, and this has occurred after each stage. Ventilator-dependence has become an issue after stage-one, and poor ventricular function, atrioventricular valve regurgitation, and pulmonary arteriovenous fistulae are the issues after stages two and three. Protein-losing enteropathy is an indication for heart transplantation that is unique to stage-three Fontan circulation.

In the current era, therefore, transplantation has become secondary or salvage therapy for somewhat older infants and children who have failed to progress through staged-reconstruction or who are failing Fontan’s physiology. In addition, infants and children with congenital heart anomalies who have experienced a variety of surgical or catheter-based interventions ending in systemic or bi-ventricular failure, arrive at transplantation, not infrequently, by means of mechanical circulatory assistance. Transplantation in this “back-up” role for treatment of congenital heart disease has produced less impressive operative and late survival, particularly among children who are failing Fontan’s physiology or who have become older following palliative therapy [17-20]. The reasons for reduced transplant operative survival seems apparent, as the poor general status of potential recipients and as the complexities of transplant-reconstruction are considered. Reduced late post-transplant survival usually relates to pre-transplant recipient sensitization, and hence, to the presence of graft-specific allo-antibodies [21, 22].

### Transplantation and the Hybrid Procedure for Palliation of Hypoplastic Left Heart Syndrome

**Fig. (12):**
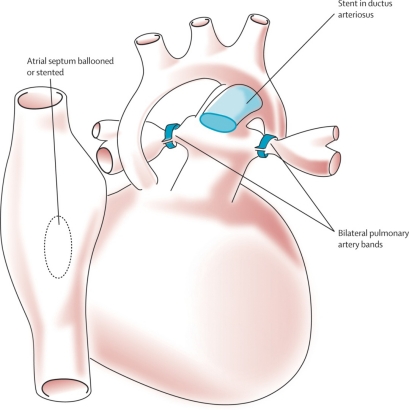
Hybrid procedure, an alternate approach to the first-stage Norwood operation. The hybrid procedure consists of surgically applying branch pulmonary artery bands and catheter-based application of a stent in the arterial duct. A balloon atrioseptostomy or insertion of an atrioseptal stent is also accomplished. Barron DJ, Kilby MD, Davies B, Wright JGC, Jones TJ, Brawn WJ. Hypoplastic left heart syndrome. Lancet 2009;374:551-64. Used with permission of the publisher.

A more recent innovation in the management of HLHS is the so-called hybrid approach. It consists of bilateral pulmonary artery banding, accomplished surgically via sternotomy, with either simultaneous or delayed catheter-based stenting of the patent ductus arteriosus. This is coupled with balloon atrioseptostomy (Fig. **12**). 

This approach has received considerable attention in Columbus, Ohio [23] and among a few other centers with hybrid laboratories which are designed to permit simultaneous high-resolution imaging, cardiac catheterization, and cardiac surgery [24, 25]. While the approach has produced reasonable outcomes, it has been slow to “catch-on,” perhaps because it requires careful patient selection and considerable operator experience to compete with outcomes following the now “standard” stage-one Norwood procedure. In addition, it adds important complexity to the second stage of reconstruction. This hybrid form of palliation may, however, have a place in stabilizing the neonate with HLHS who is on a transplant trajectory, and whose wait for a donor heart is prolonged. Removal of the ductal stent and augmentation of the branch pulmonary arteries should add little to a transplant procedure. This sequence has not yet been accomplished at Loma Linda. The Loma Linda group has, however, employed ductal stenting alone for temporary palliation on five occasions. Four of these infants experienced successful transplantation. Indeed, any discussion of hybrid palliation as bridge to transplantation remains interesting, but speculative.

### Donor Issues

Infants awaiting heart transplantation face the highest waiting list mortality of any age group listed for heart transplantation [26]. Approximately 1 in 4 infants will die while waiting, with highest mortality in infants < 3 kg, those on extracorporeal membrane oxygenation or ventilator support, requirement for prostaglandin, those with congenital heart disease for which alternate surgical palliation was not performed and non-white race/ethnicity [27]. An adequate donor supply, therefore, is one of the most important issues affecting overall mortality and has long been an issue for infant heart transplantation [28]. Indeed, the lack of a suitable donor was part of the rationale for using a baboon donor in 1984 [29].

Two relatively recent public health efforts, i.e. changes in sleep position to reduce sudden infant death syndrome (SIDS) [30] and mandatory infant car seats have potentially affected the number of donors from these sources. Child safety seats reduce the risk of death in passenger cars by 71% for infants, and by 54% for toddlers ages 1 to 4 years [31]. It is possible that this has affected the availability of donors suitable for infants, but this has not been evaluated to date.

The incidence of SIDS has been dramatically reduced in the last 15 years [30]. Still, SIDS is the third leading cause of death for infants with an estimated annual incidence in the United States of 0.54 per 1,000 live births [32]. There have been concerns regarding the use of donor hearts from infants with SIDS because of the uncertainty whether the cause of death may be cardiac in origin and then subsequently manifest itself in the recipient. A recent retrospective report utilizing data from the Unites States Organ Procurement Transplant Network data did not demonstrate a difference in clinical outcome between infants transplanted with a donor who died from SIDS versus donors who died of other causes. Interestingly, they also noted that proportion of transplanted infant donor hearts where that donor cause of death was SIDS has increased from just over 4% in 2000-02 to nearly 9% in 2006-08. This is likely due to transplant centers’ increasing confidence with the use of SIDS donors.

Cardiac transplantation in infancy using ABO incompatible donors in the appropriate setting [33] has now become an established protocol in many centers around the world. Outcomes for infants who receive an ABO incompatible donor are equivalent to those who receive a compatible donor [34, 35]. A strategy utilizing listing for ABO incompatible donation is associated with a higher likelihood of transplantation within 30 days for infants with blood group O [36]. Infants, and even some toddlers, transplanted using this protocol do not develop isohemagglutinin reactions to the “incompatible” donor blood group antigen. Interestingly, and also potentially very importantly, infants and young children transplanted with an ABO-incompatible graft have been shown to have a reduced development of antibodies specific for class II HLA, including donor specific antibodies [37]. ABO-incompatible transplantation appears “non-inferior” to ABO compatible transplantation, and in fact may have important immunologic advantages.

Donation after Circulatory Determination of Death (DCDD) has potential to impact the donor supply [38]. In a recent study, 4.3% of patients in a Neonatal Intensive Care Unit who were withdrawn from life support would have been suitable heart donors after circulatory determination of death [39]. This practice, however, has significant ethical and legal implications [40]. Currently it seems most prudent for any program using a DCDD process to do so only under the auspices of an Institutional Review Board protocol.

A more fruitful source of potential donors might be those potential donors who are not used for transplantation. Between 2000 and 2008, nearly 35% of pediatric donors in the UNOS database were not used for transplantation. It is possible that the smaller recipient pool could make it likely that there wouldn’t be a suitable recipient for every given donor. However, there are many donors turned down for reasons of presumed unsuitability of the graft. A series of 29 pediatric heart transplant procedures, utilizing grafts that had been turned down because of presumed graft unsuitability, was shown – even in the face of prolonged ischemic time – to not affect transplant survival [41].

Increasing the donor pool is in many ways more important than what occurs after transplant, for the mortality prior to transplantation in infants is higher than one year mortality after transplantation. It appears that routine listing for ABO-incompatible donation in the appropriate setting and a willingness to consider donors that appear to be unsuitable should be routine practice in pediatric heart transplant centers.

### Immunological Advantage for Younger Recipients?

One of the earliest experiments in transplantation, conducted by Billingham and Medawar [42]. demonstrated that exposure of the neonatal mouse to alloantigens induced permanent and specific abrogation of immune responsiveness to those antigens. This so-called neonatal “window of opportunity” continues to intrigue immunologists, but it is still unknown how clear or opaque a window this is.

The immune reaction to allotransplantation has been described as universal, rapid and severely destructive [43]. And, in the absence of immunosuppression, even newborn recipients may react to their graft in this fashion. Still, there appears to be an advantage to newborn transplantation. Our experience is that newborn recipients have better survival, less rejection and less cardiac allograft vasculopathy as illustrated in Figs **2**, **4** and **5**. Newborn “tolerance” is probably multi-factorial, and has been the subject of many theories. Is there negative selection, as occurs in natural self-tolerance? [44] Or, is the newborn immune system less capable of presenting antigens because of decreased expression of major histocompatibility complex (MHC) class II antigens [45]. What we do know is that delivery of blood group antigens early in life can induce tolerance specific to isohemagglutinins [46]. This occurs by elimination of donor-reactive B lymphocytes and depends upon a continued expression of antigen.

The infant who has undergone heart transplantation, with concomitant removal of the thymus, may provide important clues to the interplay between the thymus and immunosuppression in the immature human. The T-cell receptor diversity in the blood of infants who undergo heart transplantation during infancy has been shown to be markedly diminished. The normal person has approximately one billion different T cells. Some infant heart transplant recipients have as few as 1000. In addition, thymic function, as deduced from levels of human herpesvirus 7 was notably impaired [47].

There has been an increased risk of infections in infant heart transplant recipients, even late after transplantation. Interestingly, an increased risk of autoimmune disease was also noted [48].

The infant heart transplant recipient is likely to provide important clues to the development of accommodation and/or tolerance, and they should be studied with increased vigor.

## CONCLUSION

Heart transplantation during infancy has now been successfully performed for 25 years. Much has been learned, but much remains to be learned. Since a lifespan after infant heart transplantation is potentially much longer than for an adult recipient, it is imperative to more aggressively pursue methods to prevent and treat those long-term morbidities most likely to impact longevity – cardiac allograft vasculopathy, post-transplant lymphoproliferative disease, and long-term kidney function. Not noted in this review, but addressed elsewhere in this issue of the journal, is the challenge of non-adherence, particularly during adolescence.

Surgical techniques have been refined, and the debate about staged repair versus transplantation for HLHS and equivalent lesions has, because of improved results with staged repair and continued lack of sufficient numbers of suitable donors, been settled in favor of staged repair. Transplantation as primary therapy is now reserved for patients with severe valve regurgitation and/or significantly impaired ventricular function. Many of these patients, however, will come to transplantation after any of the repair stages. And, these patients, because of chronic illness and increased anti-donor antibodies, are more challenging to transplant.

The lack of suitable donors continues to limit the full expression of what could be superior therapy for many infants. Salutary public health initiatives, such as the change in infant sleep positions and mandatory infant car seats, have decreased the number of suitable donors. But, there are still many donors who are not used due to concerns, which may not be valid, regarding their suitability for transplantation.

Lastly, there is an apparent survival advantage for the youngest recipients. The graft half-life for newborn heart transplant recipients is still undetermined even 25 years later. Part of this advantage appears to be immunological. Transplantation for infants in the setting of ABO-incompatibility is possible because of their lack of pre-existing isohemagglutins, and leads to tolerance for the “incompatible” antigen. A preliminary picture is developing of the changes that occur in infants who are transplanted early in life, and who have removal of the thymus and then introduction of T-cell depleting agents and immunosuppression. But, there is still a conspicuous lack of understanding of just what might be other immunological factors involved in the so-called “neonatal window of opportunity”.

Apart from the more widespread introduction of safer mechanical assist devices in this population, it is unlikely that major changes will occur in surgical management. The current frontier for infant heart transplantation will be in the management of the sensitized recipient, improved prevention and management of long-term complications, and a better understanding of the immunologic milieu.

## Figures and Tables

**Table 1:** Distribution of Patients by Chronic Kidney Disease Category.

Chronic Kidney Disease Category	i-GFR mL/min/1.73m^2^	Number of Patients
1	>=90	22 (24%)
2	60-89	42 (45%)
3	30-59	23 (25%)
4	15-29	6 (6%)
5	<15	0 (0%)
	Total	93 (100%)

## ABBREVIATIONS

CAV: = Cardiac Allograft Vasculopathy
DCDD: = Donation after Circulatory Determination of Death
EBV: = Epstein Barr Virus
HLHS: = Hypoplastic Left Heart Syndrome
i-GFR: = Isotopic Glomerular Filtration Rate
ISHLT: = International Society for Heart and Lung Transplantation
PTLD: = Post-Transplant Lymphoproliferative Disease
SIDS: = Sudden Infant Death Syndrome
